# The SPINK Protein Family in Cancer: Emerging Roles in Tumor Progression, Therapeutic Resistance, and Precision Oncology

**DOI:** 10.3390/ph18081194

**Published:** 2025-08-13

**Authors:** Zitin Wali, Neha, Anas Shamsi, Syed Tasqeruddin, Saleha Anwar

**Affiliations:** 1 Amity Institute of Biotechnology, Amity University, Noida 201301, Uttar Pradesh, India; zitinwali@gmail.com; 2 Department of Forensic Science, Faculty of Applied and Basic Sciences, SGT University, Gurugram 122505, Haryana, India; 3 Centre of Medical and Bio-Allied Health Sciences Research, Ajman University, Ajman P.O. Box 346, United Arab Emirates; m.shamsi@ajman.ac.ae; 4 Department of Pharmaceutical Chemistry, College of Pharmacy, King Khalid University, Abha 62521, Saudi Arabia; hsyed@kku.edu.sa

**Keywords:** SPINK proteins, EGFR signaling, NF-κB pathway, MAPK signaling, prognostic biomarkers, cancer

## Abstract

The serine protease kazal-type inhibitor (SPINK) family is central to the regulation of proteolytic function, the establishment of physiological homeostasis, and the development of many disease states, including cancer. Emerging research has identified that members of the SPINK family are commonly overexpressed in most malignancies and are deeply implicated in pivotal oncogenic pathways like cell growth, epithelial-to-mesenchymal transition (EMT), metastasis, and drug resistance. This review provides an in-depth examination of structural and functional characteristics of SPINK proteins and their involvement in the onset and development of multiple cancers, which include prostrate, pancreatic, and colorectal carcinomas. Significantly, SPINK proteins regulate major signalling pathways, including EGFR, NF-κB, and MAPK, highlighting their role as prognostic biomarkers and therapeutic targets. The review underscores the most recent advancements in therapeutic strategies for SPINK-related pathways and outlines the bottlenecks that have restricted their use in the clinic. By integrating current evidence, this work signals the potential of SPINK proteins as good precision oncology candidates with novel options for cancer prognosis, treatment, and management.

## 1. Introduction

Cancer is a complex disease marked by unregulated cell proliferation, tissue invasion, and the potential to spread to distant organs through metastasis. As of 2025, the global incidence of cancer is expected to rise to 35 million new cases by 2050, representing a 77% increase compared to 2022 levels. In the United States, an estimated 2,040,000 new cancer cases and 618,120 deaths are expected in 2025, with declining mortality due to early detection and treatment advancements [1]. In India, the estimated cancer incidence will increase from 1,461,427 cases in 2022 to 1,570,975 cases in 2025, reflecting a 12.8% increase. Among males, lung cancer is projected to have the highest incidence (81,219 cases), while breast cancer remains the most common in females (232,832 cases). The crude incidence rate is estimated at 100.4 per 100,000 individuals, with northeastern states showing the highest rates. Cancer-related mortality in India is also expected to rise significantly, necessitating stronger prevention, early detection, and treatment strategies [2]. Both intrinsic factors, such as genetic predisposition, and extrinsic factors, including environmental exposures, lifestyle, and infections, influence the development of cancer [3], which disrupts normal cellular homeostasis. Various cancers exhibit distinct molecular and cellular characteristics, yet they share fundamental biological capabilities that drive tumor initiation, progression, and resistance to therapy [4]. The hallmarks of cancer, developed as a unifying model to explain tumor biology, consist of several fundamental traits. These include persistent proliferative signalling, avoidance of growth inhibition, evasion of programmed cell death, unlimited replicative potential, promotion of angiogenesis, and the ability to invade tissues and metastasize [5,6]. Additionally, emerging hallmarks such as metabolic reprogramming, immune evasion, and tumor-promoting inflammation further contribute to cancer progression [7]. The tumor microenvironment (TME), composed of immune cells, fibroblasts, and neural components, plays a pivotal role in supporting these hallmarks and facilitating tumor growth [8]. Despite advancements in cancer research, therapeutic challenges remain, particularly in targeting angiogenesis and overcoming drug resistance [9]. Gaining deeper insights into cancer biology is essential for advancing more effective approaches to its prevention and therapy.

The serine protease inhibitor Kazal-type (SPINK) family plays a crucial role in various cancers, with its members exhibiting both oncogenic and tumor-suppressive properties. SPINK13 has been identified as a tumor suppressor in hepatocellular carcinoma (HCC), where it inhibits Akt phosphorylation, a key regulator of cancer cell survival, and proliferation [10]. Dysregulation of SPINK genes can impair mitochondrial function, resulting in metabolic changes that promote cancer growth. Mitochondria are essential regulators of cellular energy and oxidative stress (OS), and abnormalities in their function are frequently identified in cancer [11]. Modifications in mitochondrial DNA (mtDNA) and its copy number influence carcinogenesis by influencing oxidative phosphorylation and reactive oxygen species (ROS) generation, which are connected to cancer cell adaptability and immune evasion [12,13]. Inflammation is another important driver of tumor growth, and SPINK gene dysregulation is involved in inflammatory responses that lead to oncogenesis [14]. Chronic inflammation affects mitochondrial homeostasis, which fuels cancer growth via OS and metabolic reprogramming [15]. In addition, SPINK mutations have been linked to poor mitochondrial ion channel modulation, which influences apoptosis resistance and tumor formation [16]. The TME, which contains inflammatory cytokines and metabolic stresses, worsens mitochondrial dysfunction and promotes cancer cell survival [17]. Targeting SPINK-mediated mitochondrial pathways is a possible option for therapeutic intervention, as repairing mitochondrial integrity and reducing OS can restrict tumor growth [18]. Understanding the interactions between SPINK, mitochondrial control, and cancer metabolism is critical for creating new therapeutic methods.

The intricate link between SPINK dysregulation, mitochondrial dysfunction, and cancer growth offers novel treatment targets. Targeting SPINK-mediated pathways can help to restore mitochondrial function, reduce OS, and inhibit tumor development. Recent advances in cancer therapies have focused on manipulating mitochondrial metabolism and SPINK signalling to improve therapy effectiveness [19]. For example, small-molecule inhibitors and gene-editing innovations, like CRISPR/Cas9, provide potential ways to repair SPINK gene abnormalities and enhance mitochondrial function in cancer cells. Furthermore, drugs aiming at interrupting metabolic reprogramming, including inhibitors targeting oxidative phosphorylation and ROS scavengers, have shown promise in preclinical animals [7]. Immunotherapeutic techniques, which include checkpoint inhibitors and cancer vaccines, may benefit from incorporating SPINK-targeted methods, since mitochondrial health plays a critical role in developing anti-tumor immune responses [20]. Future research should focus on determining the specific mechanisms by which SPINK dysregulation affects cancer metabolism, as well as investigating combinatorial therapeutic options that take advantage of both metabolic and immunological vulnerabilities. Such developments may pave the path for more effective, personalized cancer treatments with better clinical results. The current article focuses on the role of SPINK in cancer therapy, its potential as a biomarker and therapeutic target, and addressing the latest developmental progression and challenges in its clinical applications.

## 2. SPINK Family and Cancer: Classification and Functional Roles

### 2.1. Overview of SPINK Family Proteins

**Figure 1:**
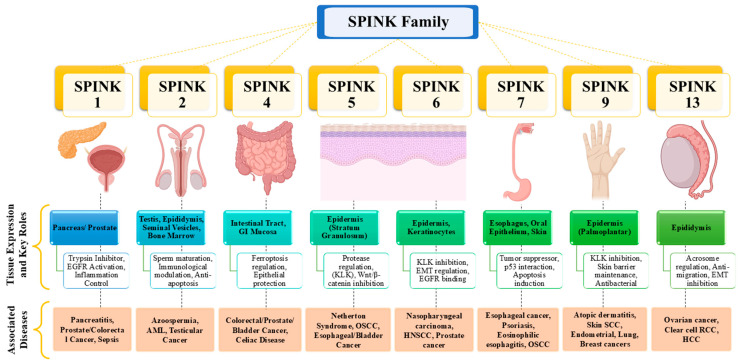
Schematic representations of tissue distribution, biological roles, and clinical relevance of SPINK family members. This schematic representation highlights the expression profiles, key physiological roles and associated diseases of SPINK family members. Each SPINK member is mapped to specific tissues or organs like pancreas, epidermis, gastrointestinal tract, and reproductive system. The diagram outlines their biological functions, including trypsin inhibition, EGFR modulation, sperm maturation, protease regulation, and epithelial protection. Pathological associations span a range of conditions, including various cancers, autoimmune disorders, and dermatological diseases like Netherton Syndrome and atopic dermatitis.

The SPINK family regulates protease activity, maintains cellular homeostasis, and influences disease development. These proteins are involved in processes such as inflammation, immune regulation, cancer progression, and tissue protection, highlighting their significance in both normal physiology and disease conditions [21,22]. Quantitative expression levels remain incompletely characterized for most SPINK proteins due to variability across tissue types and pathological conditions. The SPINK family consists of multiple members, namely SPINK 1–13, each having various tissue-specific roles (Figure 1) (Table 1) [10,14].

**Figure 2:**
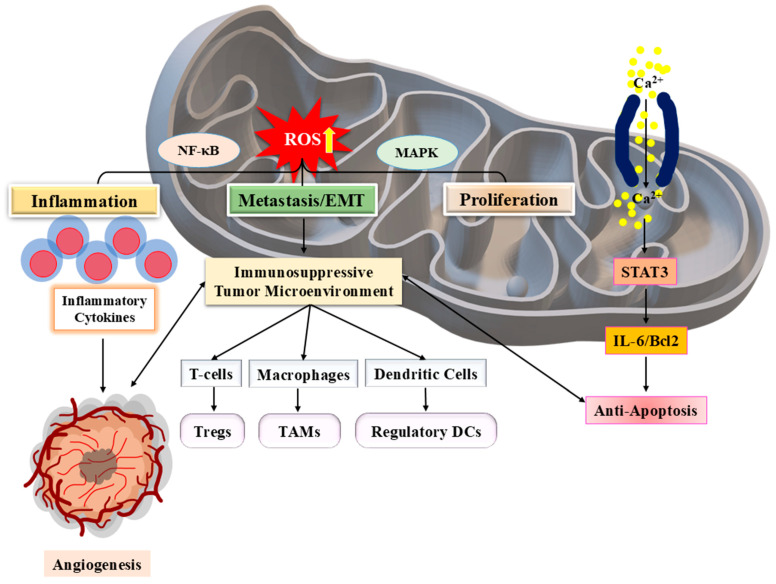
Mitochondrial dysfunction and its role in tumor progression and immune evasion. This schematic representation highlights the role of mitochondrial-induced ROS in driving key oncogenic processes, including inflammation, metastasis/EMT, proliferation, and anti-apoptosis. Elevated ROS activates NF-κB and MAPK signalling, promoting the secretion of inflammatory cytokines and supporting tumor angiogenesis. Simultaneously, mitochondrial calcium flux activated STAT3-IL-6/Bcl2 axis, leading to enhanced cell survival and resistance to apoptosis. These processes collectively contribute to the formation of an immunosuppressive tumor microenvironment by modulating immune cells like Tregs, TAMs, and regulatory dendritic cells, thereby facilitating tumor immune evasion and progression.

Most SPINK proteins are secreted serine protease inhibitors; however, secretion pattern and mechanisms may vary among family members depending on their tissue localization and physiological context. Among them, SPINK1 is the most well-studied part, extensively recognised for its role in pancreatitis, cancer, and sepsis, where it controls trypsin activity and promotes tumor growth via PI3K/Akt and MAPK pathways (Figure 2) [11,13]. It has also been found as a diagnostic and predictive biomarker for sepsis, affecting inflammatory responses and immune system regulation [21]. SPINK2 has a role in haematopoiesis and immunological modulation, as well as its transient bone marrow protease activity and apoptotic resistance [22]. In contrast, SPINK13 serves as a tumor suppressor in HCC, blocking Akt phosphorylation and lowering cancer cell growth and survival (Figure 1) [10].

Aside from their direct influence on cancer pathways, SPINK proteins interact with mitochondrial activities and OS control, particularly within TME [16,17]. Mitochondrial dysfunction, characterised by OS, metabolic reconfiguration, and mitochondrial ion channel changes, might contribute to tumorigenesis and therapeutic resistance, linking SPINK dysregulation to cancer metabolism [12,18]. Chronic inflammation, another important component in cancer progression, worsens SPINK-mediated oncogenesis by altering mitochondrial homeostasis [14,15]. Notably, SPINK mutations and mitoepigenetic alterations have been linked in breast cancer, connecting OS response to tumor growth [23].

SPINK proteins, with their various biological functions, have emerged as intriguing diagnostic markers and treatment targets for cancer, inflammatory conditions, and metabolic disorders. Targeting SPINK-related pathways, notably by modifying mitochondrial activity and lowering OS, presents promising paths for cancer treatment and precision medicine methods [18,24]. Understanding the functional variety and interactions of SPINK proteins with inflammation, mitochondrial dynamics, and TMEs is critical for establishing effective therapeutic approaches.

**Table 1:** Target Proteases, Substrates, and Functional Roles of SPINK Family Members.

S. No.	SPINK Member	Target Protease	Known Substrate	Functional Role	References
**1**	SPINK1	Trypsin, KLK5, 7	Pancreatic zymogens, Desmoglein-1 (KLK5/7)	Pancreatitis, cancer cell proliferation, Skin (Inflammation)	[25,26,27]
**2**	SPINK2	Acrosin, Trypsin-like serine proteases	Acrosomal proteins, Apoptotic regulators (Unknown)	Spermatogenesis, Apoptotic resistance in bone marrow	[22,28]
**3**	SPINK4	Trypsin, Elastase	Intestinal epithelial proteins, mucins	Colonic inflammation, intestinal homeostasis	[29]
**4**	SPINK5 (LEKTI)	KLK5, 7, 14	Desmogleins, Corneo-desmosomal proteins	Skin barrier integrity, Netherton syndrome	[30,31]
**5**	SPINK6	KLK5, 7, 14	Fibronectin, Desmosomal proteins (via KLKs)	Skin desquamation, anti-inflammatory response	[32,33]
**6**	SPINK7	Predicted trypsin-like serine protases	Unknown	Esophageal epithelial protection	[34,35]
**7**	SPINK9	KLK5	Corneo-desmosomal proteins	Palmoplantar skin barrier protection	[36]
**8**	SPINK13	Predicted trypsin-like proteases	Unknown	Tumor suppression in HCC	[10,37]

### 2.2. Role of SPINK Pathways and Different Isoforms in Cancer

Serine proteases are enzymes responsible for cleaving peptide bonds and are essential for various physiological functions, including digestion, blood clotting, and immune responses. The SPINK pathway primarily functions to inhibit these proteases through proteins containing kazal-type domains (Table 1). These inhibitors control the activity of serine proteases by attaching to them and inhibiting catalytic activity. This inhibition is required to maintain the equilibrium of proteolytic activity in biological systems [38]. To date, more than 100 kazal-type protease inhibitors have been identified; their detailed structure and function remain largely unexplored, primarily due to the high evolutionary pressure on these inhibitors, which leads to significant variability in their active sites [39].

**Figure 3:**
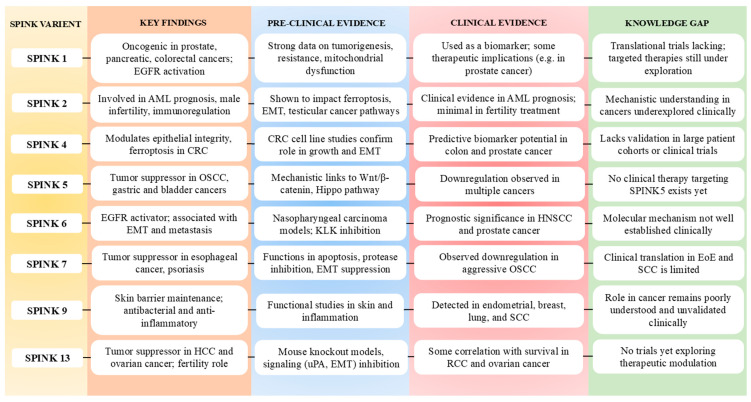
Comparative analysis of SPINK variants in cancer. A comparative analysis of oncogenic or tumor suppressive roles of various SPINK family members across different cancer types. Each SPINK variant is categorized based on key findings, pre-clinical studies, clinical validations, and existing knowledge gaps. SPINK 1 and 6 exhibit oncogenic features linking to EGFR activation and metastasis, while SPINK 5, 7, and 13 act as tumor suppressors in OSCC, HCC, and ovarian cancer. Although several variants show promising pre-clinical and biomarker evidence, many lack clinical validation, mechanistic clarity, or translational trials, highlighting significant gaps in current research and therapeutic development.

There are various types of SPINKs associated with cancer biology (Figure 3):

#### 2.2.1. SPINK1

SPINK1, also known as PST1 (Pancreatic Secretory Trypsin Inhibitor), is a protein located on chromosome 5q32, found largely in the pancreas, that regulates the activity of serine proteases, specifically trypsin. Trypsin is an enzyme that breaks down proteins in the digestive tract. Pancreatic acinar cells predominantly release SPINK1 into pancreatic juice. SPINK1 inhibits prematurely activated trypsin in the pancreas, protecting acinar cells from autodigestion and inflammation. Its primary physiological role is to inhibit early activation of trypsinogen in the pancreas. Trypsinogen is typically kept as an inactive zymogen of trypsin, although it can occasionally autoactivate itself. SPINK1 binds to and inhibits prematurely activated trypsin, preventing it from causing cell injury in the pancreas. Without functioning SPINK1, the pancreas is vulnerable to recurrent bouts of injury. Mutations in SPINK1 are linked to hereditary and tropical calcific pancreatitis [40,41,42]. The p.N34S SPINK1 mutation is associated with idiopathic pancreatitis. Beyond its role in pancreatic disorders, SPINK1 overexpression is associated with pancreatitis, Prostate cancer (PC), and chemoresistance through activation of the PI3K/Akt and MAPK pathways. Understanding the role of SPINK1 is critical for maintaining pancreatic health and avoiding pancreatitis [43]. The fusion of the ERG and TMPRSS2 genes represents the most common genetic alteration in PC. ERG functions as an oncogene encoding ETS family transcription factors, while TMPRSS2 is an androgen-responsive gene predominantly expressed in PC. Elevated SPINK1 expression serves as an independent predictor of biochemical recurrence after PC surgery, particularly in patients lacking ETS gene rearrangements. A considerable majority of primary PC patients show loss of phosphatase and tensin homolog (PTEN), albeit to different degrees. PTEN loss is strongly linked to ERG reorganization, androgen receptor (AR) amplification, and SPINK1 overexpression. Tumors with SPINK1 overexpression typically lack AR amplification and PTEN expression. PTEN functions as a tumor suppressor by dephosphorylating PIP3, thereby preventing activation of the oncogenic PI3K/Akt/mTOR-signalling pathway. The loss of PTEN activity is a common event across various types of cancer (Figure 3) [44,45,46].

Numerous loss-of-function mutations in the SPINK1 gene are strongly linked to chronic pancreatitis. These consist of promoter variations, splice-site modifications, nucleotide indels, signal peptide mutations, deletion of the initiator methionine codon, and missense variants, including the well-researched N34S mutation [47,48]. These mutations impair SPINK1 capacity to stop prematurely activated trypsin, resulting in autodigestion and long-term pancreatic damage. Crucially, chronic pancreatitis is an established precursor for pancreatic ductal adenocarcinoma, suggesting a molecular relationship between SPINK1 malfunction, chronic inflammation, and cancer formation [44]. The chronic inflammatory surroundings promote neoplastic transformation via OS, DNA damage, and altered protease activity. Thus, SPINK1 mutations not only predispose recipients to pancreatitis but also cause pancreatic carcinogenesis, emphasising its importance in diagnostics and cancer prevention.

#### 2.2.2. SPINK2

The SPINK2 gene is located on chromosome 10q22.3, which is expressed in mucosal tissues such as the skin, respiratory, and gastrointestinal (GI) tracts. By blocking the Kazal-type serine protease inhibitor, SPINK2 influences immunological responses, inflammation, and tissue remodelling. It helps to preserve epidermal barrier integrity by inhibiting desquamation-related proteases. Physiologically, SPINK2 is involved in spermatogenesis and sperm maturation by inhibiting acrosin and also contributes to hematopoiesis and immune regulation. Mutations in the SPINK2 gene are linked to Netherton syndrome, a rare genetic disease marked by severe skin abnormalities such as weakened skin barrier, a higher vulnerability to infections, and inflamed skin disorders, including eczema. These mutations may cause SPINK2 to operate less effectively, resulting in instability of serine protease function and impairment of mucosal tissue homeostasis. The expression levels of SPINK2 protein in acute myeloid leukaemia (AML) patients are studied, and their prognostic importance is analysed. Elevated SPINK2 expression has been associated with unfavourable clinical outcomes in patients with AML, suggesting its potential as an independent negative prognostic marker. Pathologically, high SPINK2 expression is associated with poor prognosis in AML and is implicated in the regulation of ferroptosis—a form of programmed cell death driven by iron-dependent lipid peroxidation [49,50].

Like all SPINKs, this also contains a characteristic kazal domain featuring six conserved cysteine residues that form three disulfide bonds, stabilizing its protease inhibitory structure. SPINK proteins block serine proteases via a classic Kazal-type binding loop, with P2-P2’ residues flanking the core P1-P1’ scissile bond. These amino acid sites regulate substrate selectivity and binding affinity of SPINK and its target proteases [51]. Its functional P2-P2’ region is composed of Pro-Arg-His-Phe residues, which are critical to its molecular activity. SPINKs bind to the protease’s active site in the same way as natural substrates would. However, the resultant SPINK–protease complex is non-cleavable and extremely persistent, thereby inhibiting the enzyme’s function [22]. SPINK2 is expressed in the testis, epididymis, and seminal vesicles, playing a vital role in maintaining sperm quality and normal reproductive function. Its shortage can cause the golgi apparatus to rupture, affecting protein processing and transport. SPINK2 levels rise in primary skin follicular centre cell lymphoma and AML, indicating a poor prognosis, which influences tumor growth and treatment results. SPINK2 is downregulated in testicular cancer, along with TIG1 (Tazarotene-induced gene 1). TIG1 and SPINK2 work together to prevent invasion, relocation, and epithelial–mesenchymal transition (EMT) of testicular tumor cells by modulating uPA/uPAR-signalling pathway, which is implicated in extracellular matrix breakdown and EMT induction [45,52,53].

#### 2.2.3. SPINK4

SPINK4 has been linked to cancer, specifically PC. SPINK1 and SPINK4 are overexpressed in some aggressive prostate tumors. These malignancies are frequently characterised by a molecular subtype referred to as “SPINK1/4-overexpressing” PC. This subtype is characterised by high-grade tumors, neuroendocrine differentiation, and resistance to androgen deprivation therapy (ADT). SPINK4 can reduce the expression of serine proteases with tumor-suppressive properties. By inhibiting the function of these proteases, SPINK4 may increase tumor development and invasion. Prostate tumors that overexpress SPINK4 are frequently resistant to standard treatments, such as ADT. This resistance might be achieved by SPINK4-induced changes in cellular-signalling pathways that favour survival and proliferation, rendering cancer cells less susceptible to therapy. It has been shown to have a crucial effect on colorectal cancer (CRC). SPINK4, by decreasing ferroptosis, may help CRC cells survive and resist treatment. Targeting SPINK4 may be a viable therapeutic option for CRC therapy. Inhibiting SPINK4 might potentially reduce CRC cell proliferation and make tumor cells more susceptible to ferroptosis-inducing drugs, enhancing therapy results for CRC patients (Figure 3) [52,54,55].

It was discovered that SPINK4 expression levels were considerably lower in CRC tissues than in surrounding normal tissues. This shows that SPINK4 may be dysfunctional in CRC, which might contribute to disease formation or progression. SPINK4 expression levels may act as a predictive factor for CRC patient outcomes. In vitro research revealed that manipulating SPINK4 expression altered CRC cell behaviours such as growth, relocation, invasion, and EMT. This shows that SPINK4 has a functional role in CRC development and metastasis [55,56,57]. SPINK4, also referred to as PEC60, is a protein derived from pig intestines that is largely expressed in the GIT and immune system. The human SPINK4 gene, situated on chromosome 9p13.3, encodes as 86-amino acid precursor protein, 60 of which are thought to be important in protecting mucosal and epithelial tissue proteins from degradation. Physiologically, it is expressed in intestinal goblet cells, where it protects epithelial barriers by inhibiting serine proteases and maintaining mucosal integrity. SPINK4 expression indicators have been found in the colon and Barrett’s oesophagus prior to the emergence of morphologically recognisable goblet cells, implying a function in detecting the initial phase of intestinal metaplasia. Furthermore, SPINK4 is also linked to bladder cancer, with increased expression associated with improved overall survival rates. The SAM tip domain ETS factor (SPDEF) regulates SPINK4, which governs the terminal distinction and maturation of intestinal goblet cells. Notch signalling has been recognised as an upstream controller of SPINK4, with suppression of Notch signalling inhibiting SPDEF and thereby downregulating SPINK4 expression [55,58,59].

#### 2.2.4. SPINK5

The SPINK5 gene, located on chromosome 5q32, codes for the lymphoepithelial Kazal-related inhibitor (LEKTI), which has 15 functional domains. SPINK5 has been recognized as a key prognostic biomarker for oral squamous cell carcinoma (OSCC). It was found to suppress the tumorigenic potential of HSC3 and SCC9 cells, while silencing SPINK5 using short hairpin RNA (shRNA) led to enhanced malignancy. The euchromatic histone lysine methyltransferase 2 (EHMT2) was shown to bind to the SPINK5 promoter, downregulating its expression. SPINK5 inhibits the Wnt/β-catenin-signalling pathway, thereby reducing aggressiveness of EHMT2-stimulated HSC3 and SCC9 cells. Treatment with Wnt/β-catenin inhibitor IWR-1 reversed the malignant phenotype in SPINK5-silenced OSCC cells. Moreover, EHMT2 silencing suppressed tumor growth and inhibited Wnt/β-catenin signalling, effects that were negated when SPINK5 was knocked down [60,61,62,63]. Inhibition of SPINK5 via EHMT2 causes OSCC. This shows that dysregulation of SPINK5 expression, possibly mediated by epigenetic changes catalysed by EHMT2, plays an important role in OSCC pathogenesis.

LEKTI regulates proteases, notably kallikrein (KLK)5 and KLK7, that are expressed in the epidermis [64,65]. SPINK5 is found in stratum granulosum close to the stratum corneum. Loss of SPINK5 activity causes NS, a disorder characterised by poor skin barrier function, which predisposes patients to a variety of skin disorders and a greater likelihood of SCC [66]. According to research, SPINK5 is considerably downregulated in oesophageal cancer, head and neck SCC (HNSCC), and bladder transitional cell carcinoma [67]. This downregulation is linked to increased tumor aggressiveness, lymphatic metastasis, and an unfavourable prognosis. SPINK5 suppresses glycogen synthase kinase-3β (GSK3β) phosphorylation in oesophageal cancer, resulting in β-catenin breakdown and reduced tumor cell proliferation, relocation, and invasion. SPINK5 induced apoptosis in gastric cancer cells by modulating BCL-2/BAX expression and NF-κB signalling. Furthermore, SPINK5 serves as a downstream target of G9a, a histone lysine methyltransferase observed in renal cell carcinoma (RCC). G9a-mediated methylation of H3K9 reduced SPINK5 expression while increasing RCC cell growth [68,69]. miR-32 inhibits SPINK5 expression in castration-resistant PC, promoting tumor development. SPINK5 suppresses the Hippo pathway in SCC, preventing the initiation of YAP1-TAZ/TEAD transcription network (Figure 3) [70]. This inhibition causes lower expression of KLK5, PAR-2, and IL-8, hence inhibiting matriptase-dependent carcinogenesis and modulating the molecular landscape of SCC formation.

#### 2.2.5. SPINK6

SPINK6, another member of the SPINK family, placed on chromosome 5q 33.1, is a strong inhibitor of epidermal proteases essential for skin homeostasis, such as KLK 5, 7, and 14. SPINK6 is a component of cluster 53 squamous epithelial cells-keratinisation, a process required for the establishment of skin barrier. SPINK6 is found in many cancer cell lines; however, the specific processes are not well known. SPINK6-associated diseases comprise Witkop syndrome and Pompholyx. SPINK6 has been isolated from human skin and shown to inhibit KLK-related peptidases selectively. SPINK6 plays a role in the intricate LEKTI proteolytic activation cascade, which inhibits certain proteinases. Unlike LEKTI, SPINK6 contains only a kazal domain. It interacts with transglutaminases in human keratinocytes and epidermis, maintaining its inhibitory activity against specific substrates of KLK-related peptidases. SPINK6 has been shown to inhibit numerous KLKs, namely, KLK4, 5, 6, 12, 13, and 14 at nanomolar to sub-nanomolar levels. SPINK6 bonds with fibronectin via transglutaminase, preventing KLK5 cleavage [71,72]. This interaction shows that SPINK6 may be involved in EMT control. SPINK6 has been found to induce EMT by binding and activating the epidermal growth factor receptor (EGFR) and downstream Akt signalling [73,74]. SPINK6 overexpression is related with poor prognosis in a variety of malignancies. It is elevated in HNSCC and PC, where it indicates the probability of mortality from HNSCC and has a role in biochemical recurrence following prostate surgery. While SPINK6 expression has been identified in colorectal cancers, its importance remains unknown, necessitating additional research [75,76].

#### 2.2.6. SPINK7

SPINK7, also found on chromosome 5q32, plays an important function to preserve skin homeostasis and is implicated in inflammatory skin disorders such as psoriasis and eczema [77]. Additionally, it has also been linked to oesophageal cancer. The SPINK7 gene, also called oesophageal cancer-related gene 2, is hypothesised to operate as a tumor-suppressor gene, controlling protease cascades in carcinogenesis and oesophageal carcinoma invasion by regulating migration via the urokinase-type plasmin activator/plasmin MAPK pathway. SPINK7 expression levels in OSCC cells were found to be significantly different from those in normal tissues. Variations in SPINK7 expression were shown to be related to tumor development and aggressiveness, indicating a role in OSCC pathogenesis. OSCC samples commonly had alterations to the HER2, p53, and RB1 genes. SPINK7 expression variations were shown to link with the state of these genes, indicating possible interaction between SPINK7 and pathways controlled by HER2, p53, and RB1 in OSCC [78].

SPINK7, also known as oesophageal cancer-related gene 2 (ECRG2), is expressed in a variety of tissues, including fetal skin, thymus, oesophagus, oral epithelium, thyroid, brain, lung, heart, stomach, liver, spleen, colon, kidney, testis, gallbladder, and adult oesophageal mucosa. However, its expression is markedly downregulated in primary oesophageal cancer tissue. Recent research suggests that SPINK7 plays an important role in maintaining skin homeostasis and is involved in inflammatory conditions such as skin disorders, bowel inflammation, eosinophilic oesophagitis, and oesophageal inflammation [35,77,79,80,81]. SPINK7 suppresses tumor development by inducing apoptosis and binding directly to the urokinase-type plasminogen activator (uPA). This interaction affects the association between the uPA receptor and β1 integrin, influencing the Src/MAPK-signalling pathway and thereby controlling cell migration and invasion. SPINK7 loss increases this interaction, stimulating the Src/MAPK pathway and promoting cancer cell motility and invasion. SPINK7 binds to uPA, which reduces proteolysis and slows cancer cell proliferation. Heteronuclear magnetic resonance studies suggest that the uPA-binding loop of SPINK7 corresponds to the serine protease reactive site loop of the third domain of turkey ovomucoid (OMTKY3), indicating a potential binding site between SPINK7 and uPA. Furthermore, SPINK7 interacts with different proteins, like metallothioneins and mitochondrial ribosomal protein S12, to regulate cell proliferation, death, and other physiological processes [35]. P53 is a crucial tumor suppressor that regulates cell growth by promoting apoptosis and facilitating DNA repair. Mutations in p53 lead to abnormal cell proliferation and tumor progression. SPINK7 localizes to centrosomes and centromeres during both interphase and mitosis, where it contributes to centrosome amplification through a p53-dependent mechanism. Loss of SPINK7 disrupts p53 stability, downregulates p21, enhances cyclinE/CDK2 activity, and impairs centrosome duplication and spindle checkpoint functions, potentially leading to chromosomal instability and aneuploidy. Additionally, when combined with cisplatin, SPINK7 reduced drug resistance in esophageal cancer by upregulating p53 and downregulating PCNA and Bcl2 expression. In OSCC, SPINK7 expression decreases, whereas p53, RB, NF-κB, and CYP4B1 increase. Severe OSCC possessed lower SPINK7 and HER2 levels but higher TP53 and RB1 activity than less aggressive OSCC. SPINK7, HER2, p53, and RB1 expression changes might be used as a biomarker to stage OSCC lesions (Figure 3) [78,82,83,84,85].

#### 2.2.7. SPINK9

With other SPINKs, SPINK9, present on chromosome 5q33.1, also protects the skin barrier by suppressing KLKs, which are enzymes responsible for skin desquamation. It regulates KLK activity, notably KLK5, preventing excessive proteolysis and preserving skin integrity [86,87]. It is mostly expressed in the epidermis, particularly in the granular layer, where it aids keratinocyte development and cornification. SPINK9 expression has been altered in atopic dermatitis patients, with lower levels associated with poor skin barrier function and higher vulnerability to inflammation. SPINK9 is also linked to cancer, with considerable cytoplasmic positivity observed in endometrial and lung malignancies, as well as in some instances of breast, renal, and gastric cancers. Furthermore, SPINK9 is highly expressed in the epidermis of human palms but is low or absent in non-palmoplantar skin. Functionally, SPINK9 inhibits KLK5 strongly, particularly under acidic conditions, and has some inhibitory impact on KLK8. It also functions as an antibacterial peptide, specifically killing *E. coli* while promoting keratinocyte migration via purinergic receptor activation and metalloproteinase/EGFR-dependent processes [88]. It is expressed in both healthy palmoplantar skin and diseases like lichen simplex chronicus, actinic keratosis, and SCC. However, its involvement in SCC is disputed. Also, SPINK9 expression is confirmed by immunohistochemical staining and molecular analysis in skin samples from individuals with these conditions. Due to KLK5’s role in cutaneous SCC, PC, and colorectal adenoma–carcinoma, SPINK9 is thought to be a tumor-promoting factor [89,90]. Understanding SPINK9 expression trends may provide medical perspective for the diagnosis, prognosis, and management of many diseases, as well as possible biomarkers or therapeutic targets. In vitro studies show that SPINK9 efficiently suppresses KLK5 activity, emphasising its function in regulating protease-mediated activities in the skin. Additional research is required to understand its role in cancer development and skin disorders.

#### 2.2.8. SPINK13

SPINK13, located at chromosome 5, plays a crucial role in sperm development and male fertility by regulating the acrosome response, preventing premature activation, and ensuring optimal sperm function during fertilization. It is predominantly expressed in the epididymis, highlighting its role in sperm maturation. Studies on SPINK13 knockout mice revealed structural abnormalities in the acrosome and sperm tail, leading to impaired motility and reduced fertility compared to wild-type mice [91,92,93,94]. Beyond reproductive functions, SPINK13 has been implicated in cancer, particularly ovarian and RCC. In ovarian cancer, SPINK13 overexpression is associated with improved survival rates, while immunohistochemical analyses show significantly lower protein levels in cancerous tissues compared to normal ones. In vitro studies demonstrate that SPINK13 inhibits cell proliferation, enhances apoptosis, and suppresses migration and EMT by downregulating uPA, a key enzyme in cancer progression. Reduced SPINK13 expression leads to elevated uPA levels, promoting invasion and metastasis. This suggests SPINK13 may serve as a tumor suppressor and potential biomarker for ovarian cancer diagnosis and prognosis. Targeted therapies aimed at increasing SPINK13 expression or inhibiting uPA activity could offer new anti-metastatic strategies [95,96,97].

SPINK13 also plays a role in RCC, particularly clear cell RCC, where its transcription and protein expression are significantly elevated. Higher SPINK13 mRNA levels correlate with reduced progression-free and overall survival rates. The protein is involved in various signalling pathways, which include complement activation, apical junction integrity, EMT, glycolysis, hypoxia, and inflammation. Mechanistically, SPINK13 interacts with uPA by modulating matrix metalloproteinases (MMPs), inhibiting MMP9 cleavage, and regulating extracellular matrix degradation. Structurally, SPINK13 contains an N-terminal signal peptide and Kazal domain. Its expression is regulated by androgens through interaction with androgen response elements [37,98,99]. Due to its tumor-suppressive properties and influence on key oncogenic pathways, further research on SPINK13’s interactions with uPA and downstream signalling mechanisms could provide valuable insights into its therapeutic potential in cancer treatment.

### 2.3. SPINK as a Biomarker and Therapeutic Target

The SPINK family has emerged as an important biomarker and therapeutic target for a variety of diseases, especially cancer and inflammatory disorders. SPINK proteins are recognised for their function in regulating protease activity, immunological responses, and TME interactions, rendering them useful markers of disease progression and possible therapeutic targets. Recent research has emphasised the diagnostic and prognostic relevance of SPINK 1, 2, 4, and 5 among other proteins in various cancers and immune-related disorders (Table 2).

SPINK1 is among the most extensively studied members of the SPINK family, particularly in the contexts of sepsis and cancer. It is recognised as a diagnostic and prognostic biomarker for sepsis, with its dysregulation influencing inflammatory pathways and immune regulation [21]. In cancer, SPINK1 overexpression is connected to tumor growth and therapeutic resistance, notably in HCC [100]. TME is essential in treatment resistance, and addressing SPINK1 in the damaged TME has been demonstrated to enhance therapeutic results, indicating the possibility as a treatment target [41]. Furthermore, genome-wide studies show that SPINK1 expression has a considerable predictive value in HNSCC, supporting its potential as a tumor biomarker [101]. Similarly, SPINK2 has been found as a prognostic biomarker in AML; its expression corresponds with immune invasion and disease progression [102]. Due to the importance of immune system regulation in leukaemia development, SPINK2’s participation in immune infiltration provides a possible target for treatment methods. Also, SPINK4 has attracted interest as a biomarker in numerous malignancies, with research confirming its relationship with survival, therapeutic response, and metastasis in pan-cancer analysis (Table 2) [103]. SPINK4 has been found in colon cancer using a KRAS gene-based signature, correlating its expression to prognosis and treatment sensitivity, indicating its importance in precision oncology [104].

Aside from its oncogenic activities, the SPINK family is important for immune control, notably in inflammation and tumor-associated immunological modulation. SPINK5 has been involved in eosinophil extracellular traps inside TME, notably in HNSCC, indicating a possible relationship between immunological dysregulation and cancer development [105]. The SPINK family, as a whole, provides a potential path for biomarker-based diagnostics and targeted treatment in cancer and inflammatory disorders.

**Table 2:** A concise overview of how SPINK serves as both a biomarker for diagnosis, prognosis, and treatment prediction, as well as a promising therapeutic target.

S. No.	Aspect	Details	Cancer Type	Reference
**1**	Diagnostic Biomarker	Elevated SPINK1 expression correlates with poor prognosis and tumor grade.	Prostate, Pancreatic	[106,107]
**2**	Prognostic Biomarker	High SPINK expression lined to aggressive tumor behavior and recurrence.	Colorectal, Ovarian	[96,108]
**3**	Predictive Biomarker	SPINK mutations predict resistance to chemotherapy and poor outcomes.	Lung, Gastric	[109,110]
**4**	Therapeutic Target	Inhibiting SPINK1 reduces tumor growth and enhances chemosensitivity.	Prostate, HCC	[10]
**5**	Role in Metabolic Pathways	SPINK—mediated mitochondrial dysfunction promotes metabolic reprogramming.	Breast, Pancreatic	[11]
**6**	Immune Modulation	SPINK overexpression facilitates immune evasion by modulating TME	Colorectal, Lung	[7]
**7**	Potential Therapeutics	SPINK inhibitors and mitochondrial modulators are under preclinical testing	Multiple Cancer Types	[19]

### 2.4. SPINK and Its Relation to Various Diseases

#### 2.4.1. Acute and Chronic Pancreatitis

SPINK 1 protects the pancreas by blocking premature trypsin activation, preventing autodigestion and inflammation. Mutations or shortcomings in the SPINK 1 gene have been shown to predispose individuals to both acute and chronic pancreatitis. It was found that heterozygous SPINK 1 impairment causes trypsin-dependent chronic pancreatitis in mice, showing that even a partial loss of SPINK 1 function can greatly enhance sensitivity to pancreatic damage [111]. Similarly, it was discovered that mice with human-relevant SPINK 1 mutation had higher incidences of chronic pancreatitis after acute episodes, highlighting the relevance of this variation in disease development [112]. The clinical significance of SPINK 1 mutations in genetic studies of patients with acute and chronic pancreatitis was studied, identifying them as substantial risk factors, particularly in idiopathic and early-onset cases [113]. A pancreas-specific AAV8-mediated delivery method for human SPINK 1 was created, which provided considerable protection against pancreatitis in mouse models with no observable toxicity, demonstrating strong proof-of-concept for gene augmentation therapy [114]. These studies show that SPINK 1 is not merely a key genetic determinant of pancreatitis susceptibility and progression but also a promising therapeutic target, with restoring or enhancing SPINK 1 function potentially offering a precise and long-lasting intervention for patients at genetic risk of this debilitating disease.

#### 2.4.2. Azoospermia

SPINK 2 is an important protease inhibitor that is involved in male fertility, specifically spermatogenesis and sperm maturation. Mutation or structural genomic alterations involving the SPINK 2 gene are now recognised as leading to non-obstructive azoospermia (NOA), a disorder defined by lack of sperm in the ejaculate owing to defective spermatogenesis. It was revealed that SPINK 2 deficiency causes severe infertility by triggering sperm head defects in heterozygotes and complete azoospermia in homozygotes, suggesting a dosage-dependent role for SPINK 2 in maintaining normal sperm development [28]. The significance of genetic assessment for SPINK 2 and related genes in the case of idiopathic NOA was emphasised, highlighting its emerging relevance in male infertility diagnosis [115,116]. Structural chromosomal aberrations, like reciprocal translocation of SPINK 2, provide credence to the idea that genomic instability impacting SPINK 2 might impair spermatogenesis pathways [117]. SPINK 2 likely protects developing germ cells by blocking proteolytic damage in seminiferous epithelium, and its absence results in apoptotic degeneration and ineffective spermatid maturation. While there are currently no clinically available gene treatments targeting SPINK 2, the potential for gene repair or substitution therapies is promising. Developments in gene editing and targeted delivery technologies may eventually restore SPINK 2 activity in afflicted testes, providing a disease-modifying therapy for particular hereditary variants of azoospermia. SPINK 2 may also act as a biomarker for early genetic diagnosis, guiding patient care and assisted reproductive technology options.

#### 2.4.3. Celiac Disease

SPINK 4 has a role in maintaining intestinal epithelial homeostasis and shielding mucosal layers from protease-mediated damage, which is especially important in the setting of celiac disease (CD), an immune-mediated enteropathy caused by gluten. SPINK family members, notably SPINK 4, are linked to CD susceptibility, with genetic variations possibly impacting epithelial barrier function and immunological responses to luminal antigens. SPINK 4 is mostly expressed in the intestinal tract, and its expression is hypothesised to control excessive protease activity during inflammation, a crucial aspect of CD pathophysiology [118]. The essential role of intestinal epithelial cells in controlling immune responses in pediatric CD was highlighted, where impaired barrier integrity facilitates aberrant T-cell activation; here, SPINK 4 is likely to serve as a protective modulator [119]. Therapeutically, targeting SPINK 4 to increase its expression or function could be a novel strategy for reinforcing epithelial defence, reducing protease-driven inflammation, and improving outcomes in CD patients, particularly those with continued symptoms, even with a gluten-free diet.

#### 2.4.4. Netherton Syndrome

SPINK 5 encodes the serine protease inhibitor LEKTI, a crucial regulator of epidermal proteolytic activity, and its deficiency is the molecular hallmark of Netherton syndrome, a rare autosomal recessive disorder characterised by ichthyosis, hair shaft defects, and atopic manifestations. Mutations in SPINK 5 disrupt LEKTI function, leading to uncontrolled activity of epidermal KLK 5 and 7, resulting in impaired skin barrier integrity, chronic inflammation, and heightened susceptibility to allergens and infections [120,121]. Intra and interfamilial phenotypic variability underscores the complexity of genotype–phenotype correlations and points towards the modifying effects of immune maturity and environmental exposures [122]. Comparative analysis in human patients and SPINK 5 knockout mice has elucidated disease-relevant pathways and reinforced the central role of LEKTI in maintaining epidermal homeostasis [123]. It was demonstrated that the inactivation of KLK 5, a downstream effector of LEKTI loss, can reverse key cutaneous symptoms in neurine models, offering a promising therapeutic avenue [124]. Additionally, the identification of novel and atypical mutations in SPINK 5 expands the mutational spectrum and supports the need for personalised diagnostic and treatment strategies. Targeting the SPINK 5 gene or its downstream effectors presents a viable therapeutic strategy, which aims to restore skin barrier function and mitigate the atopic and inflammatory phenotype of Netherton syndrome, thereby improving patient quality of life and reducing long-term complications.

#### 2.4.5. Eosinophilic Esophagitis

SPINK 7, also known as ECRG2, is being recognised as an important regulator of oesophageal epithelial barrier integrity and immunological homeostasis in eosinophilic esophagitis (EoE). SPINK 7 was found to be considerably downregulated in EoE patients, connecting its deficit to poor barrier function and persistent eosinophilic inflammation. SPINK 7 blocks serine proteases, which would otherwise destroy tight junction proteins, preserving mucosal integrity [125]. Its absence increases epithelial permeability, allowing allergen penetration and immunological activation [126]. A regulatory axis comprising aryl hydrocarbon receptor (AHR) was identified, and the transcription factor OVOL1, which, when activated, recovers SPINK 7 expression while suppressing EoE-related immunological responses [127]. Additionally, SPINK7 has been shown to function as a tumor suppressor in the DNA damage response, suggesting it may play wider protective roles in epithelial tissue biology [81]. In terms of therapy, restoring SPINK 7 levels by gene therapy, AHR agonists, or recombinant protein supplementation may reverse epithelial damage, decrease inflammation, and provide disease-modifying therapies for EoE. These data suggest that SPINK 7 might be a suitable target for future precision treatments.

#### 2.4.6. Psoriasis and Eczema

SPINK 7 is involved in the skin’s protease–antiprotease balance, which is necessary for protecting epidermal integrity and controlling inflammatory responses. It was found that SPINK 7 is expressed in the human epidermis, specifically in the granular and upper spinous layer [77]. Its expression is induced by inflammatory cytokines like IL-17A and IFN-γ, which are elevated in these conditions. It was further explained that epidermal proteolytic cascade instability leads to pathophysiology of various inflammatory skin conditions by increasing barrier breakdown, cytokine overproduction, and immune cell infiltration [128]. It was also established that SPINK 7 modulates numerous proteases to help promote inflammation resolution during wound healing, highlighting its larger involvement in immune homeostasis and tissue repair [129]. The observed increase of SPINK 7 in inflamed skin indicates a compensating defensive mechanism; however, in chronic disorders like psoriasis and eczema, this response may be inadequate or dysregulated. Enhancing SPINK 7 activity, whether by gene therapy, recombinant SPINK 7 protein delivery, or small molecule activators, is a potential therapeutic model for restoring protease homeostasis, strengthening the skin barrier and reducing inflammation.

## 3. Cancer Pathophysiology: Role of Internal and External Factors

### 3.1. Genetic and Epigenetic Alterations

**Figure 4:**
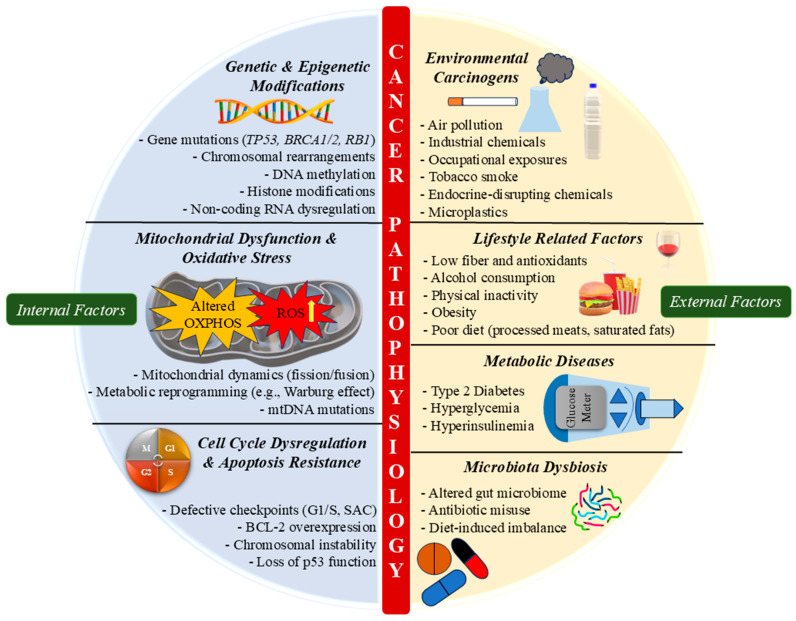
Multifactorial Drivers of Cancer pathophysiology. An illustration categorizing the diverse internal and external factors contributing to cancer pathophysiology. Internal factors include genetic and epigenetic modifications (e.g., TP53, BRCA mutations), mitochondrial dysfunction with oxidative stress, and dysregulation of call cycle leading to apoptotic resistance. These processes collectively drive tumor initiation and progression through mechanisms like altered OXPHOS, increased ROS, and loss of tumor suppressor functions. For external factors, cancer risk is exacerbated by environmental carcinogens (e.g., pollution, chemicals, tobacco), unhealthy life choices (e.g., alcohol, processed food, inactivity), metabolic diseases (e.g., diabetes and hyperinsulinemia), and microbiota dysbiosis stemming from dietary imbalance or antibiotic misuse. This integrated view highlights the need for a comprehensive understanding of both molecular and environmental components in cancer etiology.

Cancer is a complex disease driven by a combination of genetic and epigenetic modifications that influence tumor initiation, progression, and resistance to therapy. While genetic alterations involve permanent changes like mutations, insertions, and chromosomal rearrangements, epigenetic changes, which include DNA methylation, histone modifications, and regulation by non-coding RNAs, are reversible and influenced by environmental and lifestyle factors [130]. These modifications regulated gene expression without altering the DNA sequence and are increasingly recognized as critical players in cancer biology, offering diagnostic and therapeutic potential (Figure 4).

Recent studies emphasise the interplay of these factors. For example, TERT (telomerase reverse transcriptase) promoter mutations that enhance telomerase activity contribute to tumor progression in bladder carcinoma, where combined genetic and epigenetic modifications strongly impact metastasis and prognosis [131]. In GI cancers, promoter hypermethylation of specific genes has emerged as a promising biomarker for early detection and prognosis [132]. Likewise, mutations in key tumor suppressor genes such as TP53, RB1, and BRAC1/2 impair cell cycle control and DNA repair mechanisms, increasing the likelihood of malignant transformation (Figure 4) [130]. Histone modifications, mediated by histone methyltransferase (HMTs) and histone deacetylases (HDACs), regulate chromatin structure and transcription. Disruption in H3K27me3 and H3K4me3 has been associated with tumor development and therapeutic resistance [133]. In breast cancer, altered histone acetylation and methylation profiles contribute to chemoresistance by modulating the expression of drug-response genes [134]. Such epigenetic dysregulation is also implicated in SPINK-associated malignancies, influencing SPINK gene silencing or overexpression. This highlights the promise of epigenetic-targeted treatments, like HDAC inhibitors and DNA methyltransferase inhibitors, for overcoming drug resistance. As a result, targeting epigenetic alterations has emerged as a viable technique for cancer treatment, with drugs such as azacitidine and vorinostat currently licensed for haematological malignancies and being studied for other tumors. Epigenetic biomarkers have the potential to improve cancer diagnosis, prognosis, and therapeutic prediction.

### 3.2. Environmental and Lifestyle Influences

Cancer pathophysiology is shaped by the interplay of genetic, epigenetic, and lifestyle variables, which impact cancer genesis, development, and patient outcomes. Environmental carcinogens, such as air pollution, occupational exposure, and smoking, are linked to a variety of malignancies through OS, DNA damage, and chronic inflammation [135]. Likewise, interactions between the genome and environmental variables play an essential part in cancer susceptibility. Gene–environment interactions influence tumor growth and disease severity [136]. Lifestyle factors like food, physical exercise, and alcohol intake all influence cancer risk. Diets high in saturated fats and processed foods drive systemic inflammation and metabolic dysfunction, promoting tumorigenesis [137], whereas diets rich in fibre, polyphenols, and omega-3 fatty acids reduce colorectal and GI cancer risk by modulating gut microbiota and inflammatory responses. In contrast, obesity is a significant risk factor for cancers like pancreatic, breast, and colorectal, promoting low-grade inflammation, insulin resistance, and disrupted adipokine signalling [138,139], raising pro-inflammatory cytokines like tumor necrosis factor (TNF-α) and interleukin-6 (IL-6), and fostering a tumor-protecting microenvironment. Hyperinsulinemia and IGF-1 instability also contribute to malignant cell growth [140,141].

Apart from obesity, metabolic diseases like diabetes enhance cancer risk by promoting a hyperglycaemic and pro-inflammatory state, which increases OS and DNA damage. Epidemiological data suggest that individuals with type 2 diabetes are at increased risk of developing pancreatic, liver, and endometrial cancers, with hyperinsulinemia and insulin resistance playing key roles in cancer development [140]. Dysbiosis of the gut microbiota, often resulting from poor diet, pollutant exposure, or antibiotic misuse, further disrupts immune responses and fosters carcinogenic metabolite production [137]. Tobacco smoking remains a major carcinogen, triggering DNA mutations and inflammatory pathways, associated with lung, head and neck, and GI cancers [135]. Similarly, excess alcohol use increases OS, affects the DNA repair mechanism, and changes immune control. Chronic alcohol consumption is highly related to HCC, oesophageal cancer, and breast cancer. Ethanol metabolism produces acetaldehyde, a recognised carcinogen that hinders cellular integrity and increases genetic instability [142]. Physical inactivity raises cancer risk by increasing obesity, insulin resistance, and systemic inflammation [139]. Furthermore, socioeconomic factors such as healthcare access, occupational threats, and dietary costs affect cancer rates and outcomes [143]. The rising global burden of GI tumors is strongly linked to modern lifestyles. Westernised food habits, sedentary behaviours, and obesity disrupt gut microbiota and metabolism, increasing cancer risk [144]. Furthermore, emerging research shows that environmental toxins, such as endocrine-disruptive substances and microplastics, may contribute to the risk of cancer by affecting hormonal balance and immunological function [138]. Thus, cancer pathophysiology arises from a multifaceted interaction between environmental factors, genetic predisposition, and lifestyle habits. Targeted public health efforts and advances in microbiota and metabolic research can enable personalized prevention and care.

### 3.3. Inflammatory Responses and Tumor Microenvironment

Cancer pathophysiology is intricately linked to the TME, where chronic inflammation supports tumor initiation, progression, immune escape, and resistance to therapy. Pro-inflammatory cytokines, chemokines, and growth factors foster proliferation, angiogenesis, and metastasis [145]. TNF, an important inflammatory cytokine, exemplifies this dual role, promoting cell survival, immune evasion, and metastasis, in addition to possessing apoptotic properties under certain conditions [146].

In lung [147] and pancreatic cancers [148], where dense stroma and inflammatory cytokines shape tumor behaviour, SPINK expression correlates with altered immune responses and resistance to therapy. In breast cancer, inflammatory cytokine networks, especially ILs and TNF, enhance immune infiltration and metastasis [149], with SPINK overexpression contributing to EGFR and Akt pathway activation, driving EMT and tumor progression [150]. Also, in colorectal cancer, inflammation is an important promoter of carcinogenesis, with immunological dysregulation and cytokine signalling, fuelling neoplastic transformation and conventional therapy resistance [151].

IL-6, a key mediator linking inflammation to tumorigenesis, plays a critical role in PC by enhancing tumor cell survival, proliferation, and immune evasion through activation of STAT3-signalling pathway [152]. Inflammasomes further modulate immune responses and pyroptosis in TME [153], while stromal interactions, including those involving SPINK-mediated pathways, sustain a tumor-permissive state [154]. The mechanistic target of rapamycin (mTOR) pathway is now recognised as a critical regulator of immunological responses within the TME, coordinating tumor cell metabolism, immune suppression, and inflammatory signalling [155]. Aberrant mTOR activation promotes an immunosuppressive environment by changing T-cell function and enabling tumor-associated macrophage polarisation, resulting in tumor progression and resistance to treatment. Furthermore, TME remodelling by fibroblasts and immune cells hinders drug delivery, necessitating therapeutic strategies that target inflammatory cues and SPINK-associated pathways [156].

### 3.4. Dysregulation of Cell Cycle and Apoptosis

Traditionally, cellular senescence is considered a tumor-suppressive mechanism that stops proliferation of damaged or pre-malignant cells, maintaining tissue homeostasis [157]. Cellular senescence involves irreversible cell-cycle arrest, secretion of the senescence-associated secretory phenotype (SASP), macromolecular damage, and metabolic reprogramming [158]. These have been observed in pre-malignant lesions like lung adenomas [159], nevi colon adenomas, prostatic hyperplasia, and intraepithelial neoplasia [160,161,162]. Cancer cells are commonly thought to progress through the cell cycle unchecked, with malignant transformation typically requiring defects in multiple cell cycle checkpoints [163]. Despite this, they depend on intact mitotic and replication stress checkpoints, which are rarely mutated, to survive high replication burdens [164,165,166]. Cancer is marked by continuous proliferative signalling, leading to excessive and sustained cell division. Research shows that this persistent proliferation results not from uncontrolled cell cycle advancement alone but from mutations that block cell cycle exit and inhibit apoptosis [167,168,169,170,171]. Either reversibly, by starting quiescence, or non-reversibly, by senescence or death, cells can leave the cell cycle. In response to irreparable DNA damage during interphase, DNA damage checkpoints can activate quiescence, senescence, or apoptosis, primarily through p53-dependent pathways [172]. p53 mutations are among the most frequently observed genetic alterations in cancer [173]. Replication stress accelerates S-phase entry, increasing DNA damage and promoting oncogenesis via E2F-mediated feedback loops [174,175,176].

It has long been recognised that aberrant chromosomal configurations may result from mitotic errors [177], and aneuploidy is frequently seen in cancer [178]. It often arises from a malfunctioning spindle assembly checkpoint (SAC), which is common in cancer and results in chromosomal instability (CIN), which enhances tumor evolution by increasing karyotypic diversity [179,180,181]. According to recent research, cancer cells benefit from low levels of CIN, which promotes the evolution of cancer by expanding the number of potential karyotypic combinations [182,183]. However, excessive CIN can be detrimental, resulting in the loss of vital genes that cause cell death and growth inhibition [184]. A higher CIN may be linked to better patient prognosis [185].

### 3.5. Mitochondrial Dysfunction in Cancer Progression

**Figure 5:**
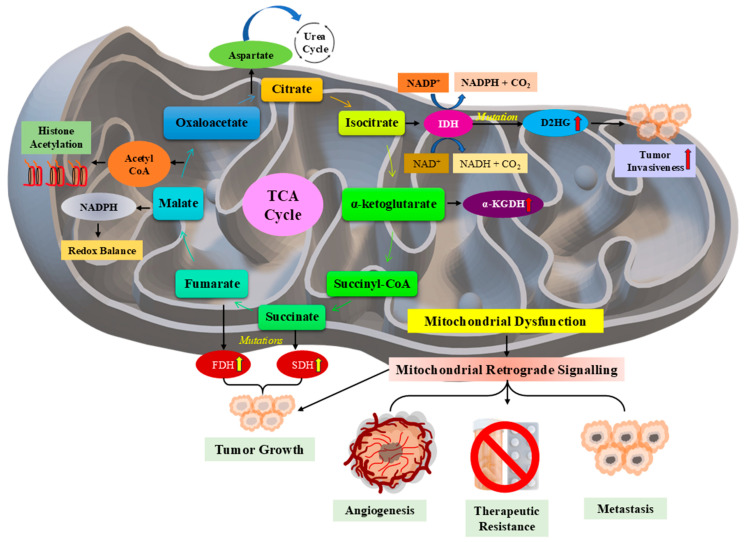
Mitochondrial Dysfunction in Cancer rogression. A schematic representation of mutations in key TCA cycle enzymes contributing to mitochondrial dysfunction and oncogenesis. Mutations in enzymes like IDH, SDH, and FH disrupt normal metabolic flux, leading to the accumulation of oncometabolites like 2-hydroxyglutarate (D2HG), succinate, and fumarate, which promote epigenetic reprogramming, tumor invasiveness, and growth. Altered levels of citrate, α-ketoglutarate, and acetyl-CoA influence histone acetylation and redox balance, further contributing to gene expression changes favorable to tumor survival. These metabolic changes initiate mitochondrial retrograde signaling, which triggers downstream effects such as angiogenesis, therapeutic resistance, and metastasis—hallmarks of cancer aggressiveness.

Mitochondrial dysfunction plays a central role in cancer progression by altering cellular metabolism, promoting OS, and influencing apoptotic resistance. As the powerhouse of the cell, mitochondria regulate energy production, biosynthesis, and redox balance; dysfunctional mitochondria contribute to tumorigenesis via metabolic reprogramming and adaptation to hypoxia (Figure 5) [186]. Hypoxia-inducible factors (HIFs) and mitochondrial dysfunction are intricately linked, shifting metabolism towards glycolysis and inducing angiogenesis, supporting tumor growth and therapy resistance [187]. Moreover, mtDNA mutations and dynamic alterations enhance tumor heterogeneity and metastatic potential by modulating bioenergetics and apoptosis evasion [188].

In addition to its role in cancer, mitochondrial dysfunction is also involved in liver conditions like nonalcoholic fatty liver disease (NAFLD) and HCC, where it promotes OS, inflammation, and proliferation (Figure 5) [189,190]. Disruptions in mitochondrial protein import are associated with neurodegenerative disorders and malignancies through impaired cellular homeostasis, which promotes tumorigenesis [191].

The Warburg effect, a key metabolic feature of cancer, exemplifies how mitochondrial alterations favor aerobic glycolysis instead of oxidative phosphorylation, even when oxygen is available. This shift supports rapid cell growth by supplying essential biosynthetic precursors while minimizing reliance on mitochondrial respiration [192]. Therapeutically, targeting this dysfunction, such as using melatonin to modulate SIRT3/PDH signalling, has shown efficacy in reversing the Warburg phenotype and restoring oxidative metabolism in cancer cells [193]. Additionally, mitochondrial dysfunction is closely linked to OS and inflammation, both of which drive cancer pathophysiology. Heme oxygenase-1 (HO-1), a central regulator of OS, can either promote or suppress tumors depending on the specific cellular environment [194]. Elevated ROS levels from dysfunctional mitochondria induce DNA damage, genomic instability, and uncontrolled proliferation. In the liver, for example, mitochondrial dysfunction exacerbates disease via OS-mediated inflammation, reinforcing therapeutic potential of restoring mitochondrial function [195].

## 4. SPINK-Mediated Signalling Mechanisms in Cancer

### 4.1. SPINK Interactions with Growth Factor Receptors

SPINK family proteins play a pivotal role in cancer progression by modulating protease activity and interacting with key growth factor receptors. Among them, SPINK 1, 6, 7, and 9 exhibit differential expression patterns across cancer types, influencing tumor growth, metastasis, and therapeutic responses. One of the primary mechanisms through which SPINK proteins drive oncogenesis is their ability to activate the EGFR pathway through direct binding to its extracellular domain, leading to receptor autophosphorylation at tyrosine residues like Y1068 and Y1173, thereby stimulating cell proliferation, migration, and survival [73,196]. SPINK 1, originally identified in pancreatic secretions, has garnered significant attention for its role in prostate, pancreatic, and CRCs. Elevated SPINK 1 expression is associated with aggressive tumor behavior and unfavourable prognosis, often functioning as an oncogene that enhances EGFR activation and downstream signalling. The interaction between SPINK proteins and EGFR is a critical driver of tumorigenic processes. SPINK 1-positive PC exhibits resistance to standard androgen deprivation therapies, highlighting the necessity of alternative treatment strategies targeting this pathway [197]. Mechanistically, SPINK 1 acts as a non-canonical EGFR ligand, triggering receptor phosphorylation, which recruits adaptor proteins like Grb2 and SOS, subsequently activating PI3K/AKT and MAPK cascades that regulate transcription, cell proliferation, and survival [198]. Similar oncogenic roles have been attributed to SPINK 6 in nasopharyngeal carcinoma, where it enhances metastasis by binding to and activating EGFR, amplifying downstream signals responsible for EMT and matrix degradation [73]. Beyond EGFR, SPINK proteins modulate metalloprotease activity, which contributes to extracellular matrix remodelling and facilitates cancer cell migration. SPINK 9, for instance, has been implicated in keratinocyte migration via metalloprotease-dependent EGFR activation, a process that may extend to epithelial cancers [196].

While SPINK proteins generally function as tumor promoters, exceptions exist within the family. SPINK 7, also known as ECRG2, functions as a tumor suppressor and plays a role in regulating DNA damage response [81]. Unlike SPINK 1 and 6, which promote oncogenesis through EGFR signalling, SPINK 7 counteracts tumor progression by enhancing genomic stability and reducing cellular proliferation. This divergence in SPINK function highlights the complexity of their signalling mechanisms and suggests that therapeutic interventions must be tailored to specific roles of SPINK proteins in different cancers. Mitochondrial dysfunction and metabolic reprogramming further intersect with SPINK-mediated pathways in cancer. The Warburg effect, marked by enhanced glycolysis and diminished oxidative phosphorylation, is a defining feature of cancer metabolism and is modulated by SPINK-mediated EGFR signalling. This occurs through downstream activation of mTORC1 by PI3K/Akt, which upregulates glucose transporter expression and glycolytic enzymes like hexokinase II, promoting anabolic metabolism and tumor cell survival. Studies suggest that mitochondrial dysfunction contributes to tumor progression by altering ROS levels and metabolic flux, thereby reinforcing oncogenic effects of SPINK 1 and 6 [186,192].

The inflammatory TME also plays a key role in SPINK-mediated oncogenesis. Chronic inflammation fosters a milieu conducive to tumor growth, and SPINK proteins interact with inflammatory cytokines to enhance cancer cell survival. For instance, SPINK1-EGFR interaction can activate NF-κB signalling via IKK complex phosphorylation, leading to increased transcription of IL-6 and anti-apoptotic genes like Bcl-2; hence, sustaining inflammation and resistance to apoptosis [147]. In CRC, for example, inflammation-driven EGFR activation is associated with SPINK 1 overexpression, contributing to enhanced tumor proliferation and chemoresistance [199]. Similarly, in HCC, mitochondrial metabolic signatures linked to SPINK–EGFR interactions influence tumor progression and therapeutic resistance [190]. Due to the centrality of these pathways, targeting SPINK-mediated signalling offers a promising avenue for cancer therapy. SPINK 1-positive cancers, particularly prostate and CRC, have been explored for targeted therapeutic interventions. The inhibition of EGFR signalling using monoclonal antibodies or small-molecule inhibitors has shown potential in mitigating SPINK 1-driven tumor growth [197]. Additionally, metabolic interventions that target mitochondrial dysfunction and OS offer an alternative approach to disrupting SPINK-driven oncogenic pathways [21,195]. Beyond SPINK 1, SPINK 6-mediated metastasis in nasopharyngeal carcinoma presents another therapeutic challenge. The use of EGFR inhibitors in combination with immunotherapies or anti-inflammatory agents could prove effective in curbing SPINK 6-induced tumor progression [73]. Therefore, SPINK-mediated signalling in cancer involves defined and receptor-specific interactions with EGFR and downstream cascades, as well as influence over inflammation, metabolism, and genomic stability, functioning as both an oncogene and tumor suppressor.

### 4.2. Impact on Cell Proliferation, Metastasis, and Therapy Resistance

Among SPINK members, SPINK 1, originally identified in pancreatic secretions, has been extensively studied for its oncogenic role across various malignancies, including prostate, breast, and GI cancers. The ability of SPINK proteins to activate EGFR signalling represents a fundamental mechanism driving tumorigenesis, facilitating uncontrolled cellular proliferation and survival [197,200]. Elevated SPINK 1 expression is associated with aggressive cancer phenotypes and unfavourable outcomes, particularly in prostate and breast cancer, where it enhances tumor invasiveness and confers resistance to standard therapies. A key oncogenic feature of SPINK 1 is its ability to sustain proliferative signalling through EGFR activation, even in the absence of canonical EGF ligands, via ligand mimicry that initiates receptor dimerization and activation. This aberrant activation leads to the stimulation of downstream pathways like PI3K/AKT and MAPK, which drive uncontrolled cell growth and survival [198,201]. In PC, SPINK 1 overexpression correlates with a distinct molecular subtype resistant to androgen deprivation therapy, underscoring its role as a driver of castration-resistant prostate cancer (CRPC) [200]. Additionally, miRNA-mediated regulation of SPINK 1 has been identified as a crucial determinant of therapy response. Specifically, miR-5089-5p has been shown to suppress SPINK 1 expression, thereby inhibiting MAPK/MMP9 signalling and reducing enzalutamide resistance in CRPC, suggesting a potential therapeutic strategy to overcome SPINK 1-mediated drug resistance [202].

Beyond proliferation, SPINK-mediated signalling significantly contributes to metastasis, a major determinant of cancer lethality. SPINK 6, for instance, has been implicated in promoting nasopharyngeal carcinoma metastasis through direct binding and activation of EGFR, which increases EMT markers like N-cadherin and Snail, reinforcing the role of SPINK–EGFR crosstalk in cancer dissemination [73]. SPINK 1 similarly enhances invasive potential by upregulating MMPs, which facilitate extracellular matrix degradation and tumor cell migration [203]. In breast cancer, a comprehensive genomic and phenotypic screening approach revealed that SPINK 1 is a critical factor in tumor invasion and survival, with high SPINK 1 expression correlating with poor patient prognosis [204]. These findings indicate that targeting SPINK 1 may offer a promising approach to curb metastasis and enhance treatment outcomes. Therapeutic resistance continues to be a formidable challenge in cancer treatment, and SPINK proteins contribute significantly to this phenomenon by modulating TME dynamics. In SPINK 1-positive PS and CRC, the damaged TME fosters a protective niche that shields tumor cells from therapeutic interventions [41]. SPINK 1-driven resistance mechanism involves the activation of survival pathways, including EGFR and NF-κB signalling, which enhance cellular resilience against chemotherapy and targeted therapies [205]. Moreover, in pancreatic cancer, SPINK 1 overexpression has been associated with increased resistance to gemcitabine, one of the primary chemotherapeutic agents used for treatment [201]. These findings highlight the necessity of developing SPINK-targeted therapeutic strategies to improve treatment responses.

### 4.3. Crosstalk with Other Oncogenic Pathways

**Figure 6:**
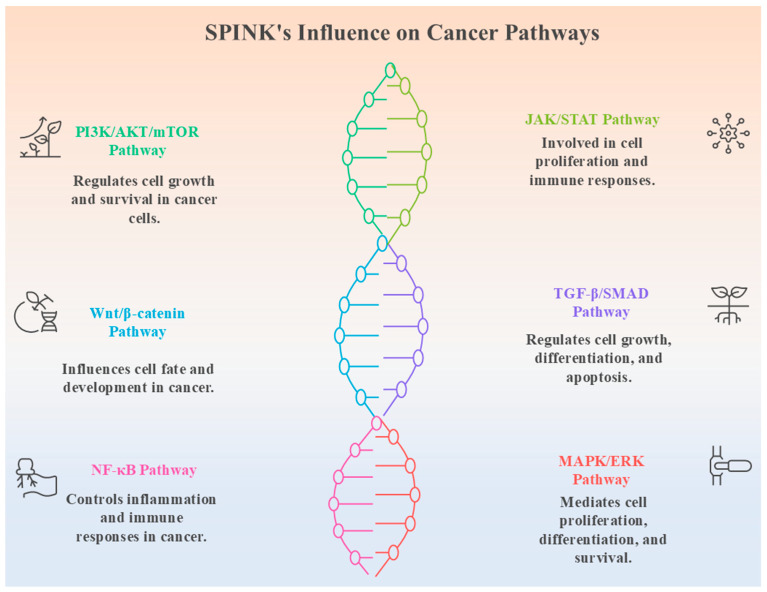
SPINK proteins and their regulatory impact on oncogenic-signalling pathways. This image highlights the pivotal role of SPINK proteins in modulating several key signalling pathways associated with cancer progression. Each pathway influenced by SPINK plays a critical role in tumour development and maintenance. PI3K/AKT/mTOR pathway promotes cell growth, metabolism, and survival, often hyperactivated in cancers. Wnt/β-catenin pathway controls cell fate, proliferation, and differentiation, crucial in cancer stem cell maintenance. NF-κB pathway regulates inflammation and immune responses, contributing to tumor-promoting microenvironments. JAK/STAT pathway facilitates cytokine signaling, enhancing tumor cell proliferation and immune evasion. TGF-β/SMAD pathway balances tumor suppression and progression, depending on the cancer stage. MAPK/ERK pathway drives cell proliferation, differentiation, and survival, frequently activated in tumors.

The SPINK family exerts their influence by modulating protease activity and orchestrating signalling networks that govern proliferation, metastasis, immune evasion, and therapy resistance. The interplay of SPINK proteins with crucial pathways, including PI3K/AKT/mTOR, JAK/STAT, Wnt/β-catenin, TGF-β/SMAD, NF-κB, and MAPK/ERK, underscores their pivotal role in tumorigenesis. SPINK family members directly inhibit specific serine proteases, predominantly trypsin and various KLKs, through their canonical kazal domains. In contrast, these various signalling molecules are not direct substrates of SPINKs. Instead, SPINKs influence these downstream pathways indirectly via upstream interactions, notably, through SPINK1’s non-canonical binding to EGFR, which can activate PI3K/Akt- and MAPK-signalling cascades [10,109]. Their role is primarily inhibitory against serine proteases, with downstream effects influencing multiple oncogenic-signalling cascades. This indirect modulation highlights the adaptor-mediated recruitment of PI3K via p85, linking EGFR engagement to broader oncogenic pathways. Understanding this crosstalk provides a framework for therapeutic interventions targeting SPINK-mediated oncogenic signalling (Figure 6).

SPINK 1 exhibits a dual function in cancer development, depending on the tumor type. In PC, SPINK 1 mutations drive aggressive phenotypes, enhancing PI3K/AKT/mTOR signalling, which is critical for cell survival and proliferation [200]. SPINK 1 upregulation has also been associated with the MECOM-SPINK 1-EGFR-signalling axis in gastric cancer, demonstrating its ability to interact with receptor tyrosine kinases and promote tumor progression [109]. This highlights the convergence of SPINK 1 with PI3K/AKT and Wnt/β-catenin pathways, where SPINK1-mediated EGFR activation leads to increased GSK-3β inhibition, resulting in nuclear accumulation of β-catenin and transcription of cyclin D1, MYC, and other oncogenes. SPINK 4 has been linked to CRC progression through its modulation of glycolysis, a metabolic hallmark of cancer [206]. Mechanistically, SPINK4-driven EGFR activation may promote glycolysis by enhancing mTORC1-mediated expression of glycolytic enzymes, suggesting an intersection between SPINK 4 activity and PI3K/AKT/mTOR pathway, which is known to regulate metabolic reprogramming in tumors. Additionally, SPINK 4 may influence the NF-κB pathway due to its established role in metabolic adaptation and inflammatory responses in TME. SPINK 5, on the other hand, exhibits tumor-suppressive properties. It inhibits oesophageal SCC metastasis by modulating immune activity, suggesting a role in immune surveillance [207]. However, epigenetic silencing of SPINK 5 via EHMT2 in OSCC leads to tumorigenesis, likely through loss of STAT1-mediated immune signalling and impaired apoptosis, implicating it in immune escape mechanisms and resistance to apoptotic signals [63]. The suppression of SPINK 5 correlates with altered JAK/STAT signalling, where decreased immune modulation contributes to an aggressive tumor phenotype. SPINK13 has been recognised as a tumor suppressor in HCC, mainly by suppressing Akt phosphorylation [10]. This has been mechanistically linked to its inhibition of upstream kinases like PDPK1 or modulation of phosphatase PTEN activity, reinforcing SPINK 13’s role in attenuating proliferative signals. Transcriptomic studies on SPINK 13 further confirm its impact on gene expression patterns associated with oncogenic signalling in HCC [208]. Since AKT phosphorylation is a central event in multiple pathways, including Wnt/β-catenin and MAPK/ERK, the functional relevance of SPINK 13 extends beyond a single signalling axis (Figure 6).

The Wnt/β-catenin pathway, a fundamental driver of tumorigenesis, is significantly impacted by the SPINK family. The regulation of this pathway by SPINK 1 has been demonstrated through its interaction with EGFR and MECOM [109]. Furthermore, EGFR-induced Akt signalling suppresses GSK3β, reducing β-catenin degradation and enabling its nuclear translocation, emphasizing the role of post-translational modifications in SPINK-mediated oncogenic crosstalk [209]. This is activated by AXIN1-295aa, a protein encoded by circAXIN1, which underscores the complexity of regulatory networks converging on this pathway [210]. TGF-β/SMAD signalling, a key determinant of EMT, is another pathway influenced by SPINKs. Low-field magnetic stimulation has been shown to accelerate oligodendrocyte precursor cell differentiation via non-canonical TGF-β signalling, revealing novel regulatory mechanisms of this pathway [211]. Although not a direct effector, SPINK-EGFR interaction may potentiate TGF-β-induced SMAD phosphorylation via ERK co-activation, hence enhancing EMT marker expression. The integration of multiomics and spatiotemporal analysis further enhances the understanding of EMT and its clinical implications in cancer progression [212]. SPINK capacity to influence EMT-related pathways indicates their role in promoting metastasis and contributing to therapy resistance (Figure 6). The NF-κB pathway, a master regulator of inflammation and survival, is also subject to SPINK-mediated modulation. Since NF-κB interacts with PI3K/AKT, JAK/STAT, and MAPK/ERK signalling, SPINK-induced EGFR activation may enhance p65 nuclear translocation, exerting a combinatorial effect on pro-inflammatory and survival gene expression. The genetic and biological drivers of PC disparities have underscored the role of inflammation-driven signalling in disease progression [213]. This suggests that SPINK-related alterations in NF-κB signalling may contribute to tumor heterogeneity and differential responses to therapy. The MAPK/ERK pathway, a critical mediator of mitogenic signals, exhibits strong interactions with SPINKs. The network diffusion-based approach for survival prediction in papillary RCC has highlighted key biomarkers linked to MAPK/ERK signalling [214]. The pharmacological properties of indirubin and its derivatives have further demonstrated the potential of targeting these pathways for cancer therapy [215]. This suggests that therapeutic strategies directed at SPINKs could leverage MAPK/ERK inhibition to counteract tumor growth.

## 5. Therapeutic Implications and Future Directions

### 5.1. Potential Strategies for Targeting SPINK in Cancer Therapy

SPINK family proteins have emerged as critical regulators of oncogenesis, influencing multiple signalling pathways that drive tumor initiation, progression, and therapy resistance. Their elevated expression has been linked to multiple cancers, which include PC, HNSCC, GI, lung, and breast, highlighting their potential as therapeutic targets [205]. Recent studies have highlighted SPINKs as diagnostic and prognostic biomarkers, with genome-wide analyses revealing their altered expression profiles in aggressive tumors [101]. Due to their intricate role in tumor biology, therapeutic strategies targeting SPINKs could provide novel avenues for cancer treatment (Table 3). One potential approach involves directly inhibiting SPINK proteins using monoclonal antibodies or small-molecule inhibitors. The oncogenic effects of SPINK 1, for instance, are mediated through its interaction with EGFR, activating downstream PI3K/AKT and MAPK/ERK-signalling pathways. Targeting SPINK 1–EGFR interactions with neutralizing antibodies or tyrosine kinase inhibitors could effectively suppress tumor growth in cancers where SPINK 1 is a known driver, such as pancreatic and PC (Table 3). Similar strategies have been employed in targeting ERG fusion-positive PC, where molecular inhibitors against specific oncogenic drivers have shown promise in preclinical models [216].

RNA-based therapies, including small interfering RNAs and antisense oligonucleotides, represent a promising strategy for silencing SPINK gene expression. RNA interference-mediated knockdown of SPINK 1 has been shown to reduce tumor growth and improve chemotherapy sensitivity in PC models. Moreover, the discovery of SPINK-associated long non-coding RNAs and microRNAs in lung and breast cancers reinforces the therapeutic promise of RNA-based approaches (Table 3). Notably, studies utilizing outlier analysis have identified TM4SF4 and LRRK2 as key oncogenic drivers, demonstrating that transcriptomic profiling can aid in discovering novel targets for personalized cancer therapy [217]. Immunotherapy is another compelling strategy due to the pivotal role SPINKs play in regulating immune responses within TME. SPINK 5, for example, has been implicated in immune suppression in HNSCC, potentially aiding in tumor immune escape [101]. Targeting SPINK5-mediated immune suppression to restore anti-tumor immunity could improve the effectiveness of immune checkpoint therapies, including anti-PD-1/PD-L1 and anti-CTLA-4 treatments (Table 3). Additionally, recent findings have revealed the therapeutic potential of SPINK 4 in colitis, suggesting that its immunomodulatory properties could be harnessed in inflammation-driven cancers, particularly in the GI tract [218]. These insights underscore the need for further exploration of SPINK-targeted immunotherapeutic strategies. Combination therapies integrating SPINK inhibitors with existing treatment modalities could also yield promising results. Tumors driven by SPINK proteins often show resistance to standard chemotherapy and targeted treatments, largely due to activation of survival pathways like NF-κB and TGF-β/SMAD. Combining SPINK inhibitors with PI3K or MAPK inhibitors may enhance treatment efficacy by preventing compensatory signalling activation (Table 3). In lung and breast cancer, where multiple oncogenic drivers coexist, rational drug combinations targeting both SPINKs and other tumor-promoting factors could improve clinical outcomes [217].

Despite these promising strategies, challenges remain in translating SPINK-targeted therapies into clinical applications. The functional diversity of SPINK family members presents a major hurdle, as some exhibit tumor-promoting properties, while others act as tumor suppressors, as seen in the case of SPINK 13 in HCC [205]. Therefore, patient stratification based on SPINK expression profiles is essential to ensure precision-targeted interventions. Advanced multiomics approaches, integrating genomics, proteomics, and metabolomics, may help identify patient subgroups that are most responsive to SPINK-targeted treatments. Moreover, resistance mechanisms to SPINK inhibition must be thoroughly investigated. As seen in ERG fusion-positive PC, where resistance to AR-targeted therapies has been a major challenge, similar adaptive responses could emerge in SPINK-inhibited tumors [216]. Identifying compensatory pathways and potential resistance biomarkers will be critical in optimizing SPINK-based treatment strategies. Advances in precision oncology, particularly in patient stratification and resistance mechanism studies, will be crucial for translating these findings into clinically viable treatments. Further research focusing on SPINK-related oncogenic networks and their interplay with tumor immune microenvironment will contribute to the development of innovative and more effective cancer therapies.

**Table 3:** Highlights both conventional and emerging strategies for targeting SPINK in cancer therapy, offering insights into potential clinical applications.

S. No.	Strategy	Mechanism of Action	Therapeutic Approach	Cancer Type	References
**1**	SPINK Inhibitors	Direct inhibition of SPINK proteins to suppress tumor growth	Small-molecule inhibitors	Prostate, Pancreatic	[10]
**2**	RNA Interference (RNAi)	Silencing SPINK gene expression to inhibit oncogenic activity	siRNA and shRNA-based therapies	Hepatocellular, Colorectal	[219]
**3**	CRISPR—Cas9 Gene Editing	Targeted deletion or correction of SPINK gene mutation	Genome-editing technology	Breast, Lung	[220,221]
**4**	Mitochondrial Modulators	Restoration of mitochondrial function altered by SPINK dysregulation	Antioxidants OXPHOS inhibitors	Breast, Pancreatic	[11]
**5**	Immune Modulation	Enhance anti—tumor immunity by targeting SPINK-induced immune evasion	Immune checkpoint inhibitors	Colorectal, Lung	[222,223]
**6**	Combination Therapy	SPINK inhibition alongside chemotherapy or targeted therapy	Dual drug regimens	Prostate, Ovarian	[224,225]
**7**	Nutritional Modulation	Diet-based approaches to reduce SPINK-mediated inflammation	Antioxidant-rich diets, supplements	Multiple Cancer Types	[226]

### 5.2. Challenges in Developing SPINK Inhibitors

SPINK family proteins are key contributors to tumor progression across multiple cancers, positioning them as promising therapeutic targets. Nevertheless, developing SPINK inhibitors faces several challenges, including their dual role in tumorigenesis, their complex interactions with oncogenic pathways, resistance mechanisms, and their delivery issues. While SPINK protein like SPINK 1 is known to drive tumorigenesis in pancreatic, PC, and CRC by activating signalling pathways like PI3K/AKT, Wnt/β-catenin, JAK/STAT, and NF-κB, other members, such as SPINK 5 and 13, have been reported as tumor suppressors in certain contexts, adding a layer of complexity to therapeutic targeting [10,205]. This functional duality necessitates precise molecular characterization before any SPINK-targeted therapy can be effectively deployed.

A major challenge in creating SPINK inhibitors lies in their involvement in activating critical oncogenic-signalling pathways, which differ across cancer types. SPINK 1, for example, promotes PC progression by interacting with EGFR, leading to downstream activation of PI3K/AKT and MAPK/ERK pathways, which enhance proliferation and survival [200]. Additionally, the MECOM-SPINK 1-EGFR-signalling axis has been identified in gastric cancer, further highlighting the relevance of SPINK 1 in aggressive tumor phenotypes. However, due to pathway redundancies, targeting SPINK 1 alone may not be sufficient, as tumors could activate compensatory pathways, such as Wnt/β-catenin and TGF-β/SMAD cascades, to sustain their malignant potential [209,210]. Another major challenge in SPINK-targeted therapy is its secretory nature, which enables it to act in an autocrine and paracrine manner, influencing not only cancer cells but also TME. This makes small-molecule inhibition difficult, necessitating the use of monoclonal antibodies or peptide-based inhibitors, which have their own pharmacokinetic limitations [101]. Furthermore, inhibition of SPINK proteins may inadvertently affect normal physiological processes, as some SPINK members, such as SPINK 5, are essential for epithelial barrier integrity and immune homeostasis [63]. Loss of SPINK 5 has been associated with increased metastasis in oesophageal SCC due to immune dysregulation [207], highlighting the importance of targeted delivery methods to reduce potential side effects.

The involvement of SPINKs in metabolic and immune regulation further complicates therapeutic strategies. SPINK 4 has been linked to glycolysis modulation in CRC, suggesting that metabolic adaptations could influence therapeutic response [206]. Additionally, SPINK proteins can influence immune responses within tumors, as demonstrated by SPINK 5’s role in immune suppression and SPINK 4’s involvement in regulating colitis [218]. Thus, the development of SPINK inhibitors must account for these immune—metabolic interactions to avoid exacerbating inflammation or promoting immune evasion. An ongoing challenge is the discovery of dependable biomarkers to enable effective patient stratification. Given the heterogeneity of SPINK expression across different cancers, transcriptomic and proteomic analyses are essential for selecting patients most likely to benefit from such therapies. Network—based approaches integrating multi—omics data have been employed to identify predictive biomarkers in papillary RCC and could be extended to SPINK—driven malignancies [214]. However, the lack of large—scale clinical studies validating SPINK expression as a prognostic or predictive marker remains a limitation.

### 5.3. Emerging Trends in Cancer Therapy

Cancer treatment has evolved significantly over past decades, transitioning from conventional chemo and radio therapies to highly sophisticated personalised medicine approaches. The advent of precision oncology has enabled treatments tailored to individual genetic and molecular profiles, significantly improving patient outcomes [227]. Biomarker, immune and RNA-based strategies have reshaped the therapeutic response, offering novel avenues for targeted intervention [228]. However, challenges remain in optimizing efficacy, overcoming resistance and ensuring accessibilities to these advanced treatments.

Personalized medicine has gained prominence with the identification of predictive biomarkers that help stratify patients for targeted therapies. Advances in liquid biopsy techniques, next generation sequencing and multi-omics analyses have enabled real-time monitoring of tumor evolution and therapeutic response [228]. This approach has been particularly effective in immunotherapy, where predictive biomarkers such as PD-L1 expression, tumor mutational burden and microsatellite instability guide the use of immune checkpoint inhibitors [229]. Personalized vaccines based on neoantigens have also shown great promise, leveraging tumor-specific antigens unique to each patient to trigger strong immune responses [230]. The role of RNA-based therapeutics in cancer treatment is rapidly expanding, particularly in immunotherapy. mRNA vaccines, initially developed for infectious diseases, have shown promise in generating anti-tumor immunity by delivering tumor-specific antigens to antigen-presenting cells [231]. These vaccines are under investigation for several cancers, like glioblastoma and ovarian cancer, and hold potential to transform cancer immunotherapy by generating long-lasting immune responses [231,232]. Furthermore, miRNA-based therapies have gained attention for their ability to regulate oncogenic pathways and sensitize tumors to existing treatments [233]. However, challenges such as stability, efficient delivery, and off-target effects need to be addressed to enhance their clinical translation [234].

The integration of artificial intelligence (AI) in cancer therapy has further accelerated the shift towards precision medicine. AI-driven algorithms facilitate early diagnosis, treatment response prediction, and drug discovery, thereby optimizing therapeutic decision-making. AI-driven analysis of large-scale genomic data has facilitated the discovery of new drug targets and the development of personalized treatment plans, reducing reliance on trial-and-error methods in cancer therapy [235]. Despite these advancements, ethical considerations, data privacy concerns, and algorithm biases must be addressed to ensure the responsible use of AI in clinical settings. Immune checkpoint inhibitors, T cell-based therapies, and cancer vaccines continue to shape the future of immunotherapy. The combination of immune checkpoint blockade with other immunomodulatory agents has demonstrated synergistic effects, overcome resistance mechanisms, and improved patient response [236]. Additionally, engineered T cells, such as chimeric antigen receptor T cells, have achieved remarkable success in hematologic malignancies, and efforts are underway to extend their efficacy to solid tumors [237]. The use of nanotechnology in immunotherapy has also emerged as a promising avenue, enabling targeted drug delivery, enhanced antigen presence, and reduced systemic toxicity [238]. Therefore, future studies should prioritize the development of affordable treatment strategies, the optimization of combination therapies, and the integration of real-world data to enhance and personalize treatment protocols. As cancer therapy continues to evolve, the integration of multi-disciplinary approaches, including genomics, immunotherapy, RNA-based treatments, and AI-driven precision medicine, holds potential to transform patient outcomes. The convergence of these fields will pave the way for highly personalized and effective therapeutic interventions, ultimately improving survival rates and quality of life for cancer patients worldwide.

## 6. Conclusions

SPINK proteins have been recognized as significant contributors to the progression of cancer, influencing proliferation, metastasis, EMT, and drug resistance. By modulating critical pathways like EGFR, NF-κB, and MAPK, they are highly promising as therapeutic targets and prognostic indicators. While the current evidence emphasizes their oncogenic potential, further investigation is needed to unravel their regulatory mechanisms and translate such information into effective clinical interventions. SPINK-targeted therapies can potentially generate new possibilities in precision oncology since they could enhance cancer prognosis, diagnosis, and therapeutic response.

## Data Availability

No data were used for the research described in the article.

## Abbreviations

ADT	Androgen Deprivation Therapy
AHR	Aryl Hydrocarbon Receptor
AML	Acute Myeloid Leukemia
AR	Androgen Receptor
CD	Celiac Disease
CIN	Chromosomal Instability
CRC	Colorectal Cancer
CRPC	Castration Resistant Prostate Cancer
ECRG2	Esophageal Cancer-related Gene 2
EGFR	Epidermal Growth Factor Receptor
EHMT2	Euchromatic Histone Lysine Methyltransferase 2
EMT	Epithelial–Mesenchymal Transition
EoE	Eosinophilic Esophagitis
GI	Gastrointestinal
GSK3β	Glycogen Synthase Kinase 3 beta
HCC	Hepatocellular Carcinoma
HDAC	Histone Deacetylase
HIF	Hypoxia Inducible Factor
HMT	Histone Methyltransferase
HNSCC	Head and Neck Squamous Cell Carcinoma
HO1	Heme Oxygenase 1
IL	Interleukin
KLK	Kallikrein
LEKTI	Lymphoepithelial Kazal-type-related Inhibitor
MMP	Matrix Metalloproteinase
mtDNA	mitochondrial DNA
mTOR	Mechanistic Target of Rapamycin
NAFLD	Non-Alcoholic Fatty Liver Disease
NOA	Non-Obstructive Azoospermia
NS	Netherton Syndrome
OMTKY3	Turkey Ovomucoid Third Domain
OS	Oxidative Stress
OSCC	Oral Squamous Cell Carcinoma
PC	Prostate Cancer
PST1	Pancreatic Secretory Trypsin Inhibitor
PTEN	Phosphatase and Tensin Homolog
RCC	Renal Cell Carcinoma
ROS	Reactive Oxygen Species
SAC	Spindle Assembly Checkpoints
SASP	Senescence Associated Secretory Phenotype
SCC	Squamous Cell Carcinoma
shRNA	Short Hairpin RNA
SPINK	Serine Protease Inhibitor Kazal Type
TERT	Telomerase Reverse Transcriptase
TIG1	Tazarotene-induced Gene 1
TME	Tumor Microenvironment
TNF	Tumor Necrosis Factor
uPA	Urokinase-type Plasminogen Activator
